# Comparison of the catalytic activity for the Suzuki–Miyaura reaction of (η^5^-Cp)Pd(IPr)Cl with (η^3^-cinnamyl)Pd(IPr)(Cl) and (η^3^-1-*t-*Bu-indenyl)Pd(IPr)(Cl)

**DOI:** 10.3762/bjoc.11.269

**Published:** 2015-12-08

**Authors:** Patrick R Melvin, Nilay Hazari, Hannah M C Lant, Ian L Peczak, Hemali P Shah

**Affiliations:** 1 The Department of Chemistry, Yale University, P. O. Box 208107, New Haven, Connecticut, 06520, USA

**Keywords:** cross-coupling, homogeneous catalysis, NHC ligands, palladium, Suzuki–Miyaura reaction

## Abstract

Complexes of the type (η^3^-allyl)Pd(L)(Cl) and (η^3^-indenyl)Pd(L)(Cl) are highly active precatalysts for the Suzuki–Miyaura reaction. Even though allyl and indenyl ligands are similar to cyclopentadienyl (Cp) ligands, there have been no detailed comparative studies exploring the activity of precatalysts of the type (η^5^-Cp)Pd(L)(Cl) for Suzuki–Miyaura reactions. Here, we compare the catalytic activity of (η^5^-Cp)Pd(IPr)(Cl) (IPr = 1,3-bis(2,6-diisopropylphenyl)-1,3-dihydro-2*H*-imidazol-2-ylidene, **Cp**) with two commercially available catalysts (η^3^-cinnamyl)Pd(IPr)(Cl) (**Cin**) and (η^3^-1-*t*-Bu-indenyl)Pd(IPr)(Cl) (**^tBu^Ind**). We show that **Cp** gives slightly better catalytic activity than **Cin**, but significantly inferior activity than **^tBu^Ind**. This order of activity is rationalized by comparing the rates at which the precatalysts are activated to the monoligated Pd(0) active species along with the tendency of the starting precatalysts to comproportionate with monoligated Pd(0) to form inactive Pd(I) dimers. As part of this work the Cp supported Pd(I) dimer (μ-Cp)(μ-Cl)Pd_2_(IPr)_2_ (**Cp^Dim^**) was synthesized and crystallographically characterized. It does not readily disproportionate to form monoligated Pd(0) and consequently **Cp^Dim^** is a poor catalyst for the Suzuki–Miyaura reaction.

## Introduction

**Figure 1:**
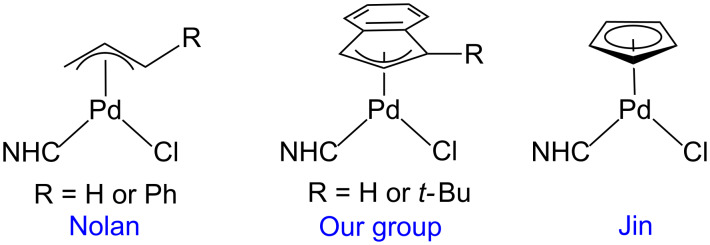
General depictions of allyl and related precatalysts that are highly active for the Suzuki–Miyaura reaction with NHC ligands.

The Suzuki–Miyaura reaction is a powerful synthetic method for forming C–C bonds between aryl halides or pseudo halides and organoborane containing species [1–5]. The most active catalysts are generally based on Pd and feature strongly electron-donating and sterically bulky phosphine or N-heterocyclic carbene (NHC) ancillary ligands [6–7]. In particular, precatalysts of the type (η^3^-allyl)Pd(NHC)(Cl) have shown excellent activity for the Suzuki–Miyaura reaction, with systems incorporating an η^3^-cinnamyl moiety giving the best catalytic results (Figure 1) [8–12]. Recently, we showed that the excellent activity of the cinnamyl system is related to two factors: (i) the rate at which the Pd(II) precatalyst is reduced to the active monoligated Pd(0) species; and (ii) the difficulty of comproportionation between L-Pd(0) and the starting precatalyst, which generates a Pd(I) μ-cinnamyl dimer of the form (μ-cinnamyl)(μ-Cl)Pd_2_(L)_2_, and removes L-Pd(0) from the reaction mixture [13–14]. Furthermore, we used this mechanistic information to design an improved precatalyst scaffold featuring an η^3^-indenyl ligand [15]. In particular, precatalysts based on the (η^3^-1-*t*-Bu-indenyl)Pd(L)(Cl) scaffold were highly active because Pd(I) dimer formation was effectively suppressed and the rate of reduction from Pd(II) to Pd(0) was increased [16].

In organometallic chemistry, allyl and indenyl ligands are considered to be closely related to cyclopentadienyl (Cp) ligands [17]. Nevertheless, to the best of our knowledge there are only two reports describing the catalytic activity of complexes of the type (η^5^-Cp)Pd(NHC)(Cl) for the Suzuki–Miyaura coupling, as well as related cross-coupling reactions [18–19]. These preliminary reports indicate that (η^5^-Cp)Pd(NHC)(Cl) precatalysts are highly active. For example, full conversion at room temperature was achieved using simple aryl chlorides as the substrate in Suzuki–Miyaura couplings at relatively low catalyst loadings (1 mol %) [18]. However, despite this impressive activity, a direct comparison of the performance of (η^5^-Cp)Pd(NHC)(Cl) type precatalysts with the related commercially available (η^3^-allyl)Pd(NHC)(Cl) and (η^3^-indenyl)Pd(NHC)(Cl) systems under the same reaction conditions has never been performed. Here, we directly assess the activity of (η^5^-Cp)Pd(IPr)(Cl) (IPr = 1,3-bis(2,6-diisopropylphenyl)-1,3-dihydro-2*H*-imidazol-2-ylidene, **Cp**) to the analogous (η^3^-cinnamyl)Pd(IPr)(Cl) (**Cin**) and (η^3^-1-*t*-Bu-indenyl)Pd(IPr)(Cl) (**^tBu^Ind**) precatalysts [20]. We show that the performance of **Cp** fits into our model of precatalyst performance based on the speed at which a scaffold is reduced from Pd(II) to Pd(0) and its tendency to undergo comproportionation.

## Results and Discussion

### Catalytic comparison of (η^5^-Cp)Pd(IPr)Cl, (η^3^-cinnamyl)Pd(IPr)Cl and (η^3^-1-*t*-Bu-indenyl)Pd(IPr)Cl

**Scheme 1:**
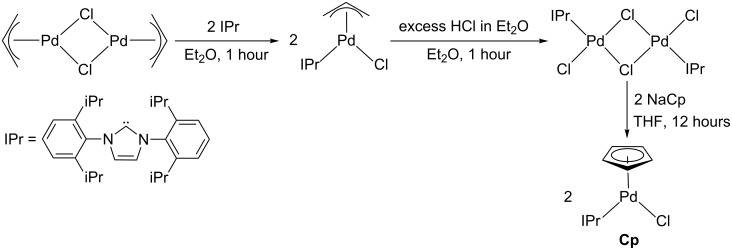
Synthesis of **Cp**.

The IPr supported precatalyst for the Suzuki–Miyaura reaction **Cp** was synthesized using a literature method starting from the commercially available Pd(II) dimer (μ-Cl)_2_Pd_2_(η^3^-allyl)_2_ (Scheme 1) [18]. It is notable that in this synthesis dimeric {(IPr)Pd(Cl)}_2_(μ-Cl)_2_ is prepared as an intermediate, followed by treatment with two equivalents of NaCp to generate the monomer **Cp**. This synthesis makes rapid ligand screening using the Cp supported scaffold difficult as the Cp group is introduced after the ligand. In contrast, the syntheses of both **Cin** and **^tBu^Ind** involve the initial preparation of dimers of the form {(η^3^-cinnamyl)Pd}_2_(μ-Cl)_2_ or {(η^3^-1-*t*-Bu-indenyl)Pd}_2_(μ-Cl)_2_, respectively [11,15], which can then be treated with a ligand to generate the ligated precatalyst. Despite repeated attempts we were unable to synthesize a related unligated Cp containing dimer, which could be used for ligand screening [21].

**Figure 2:**
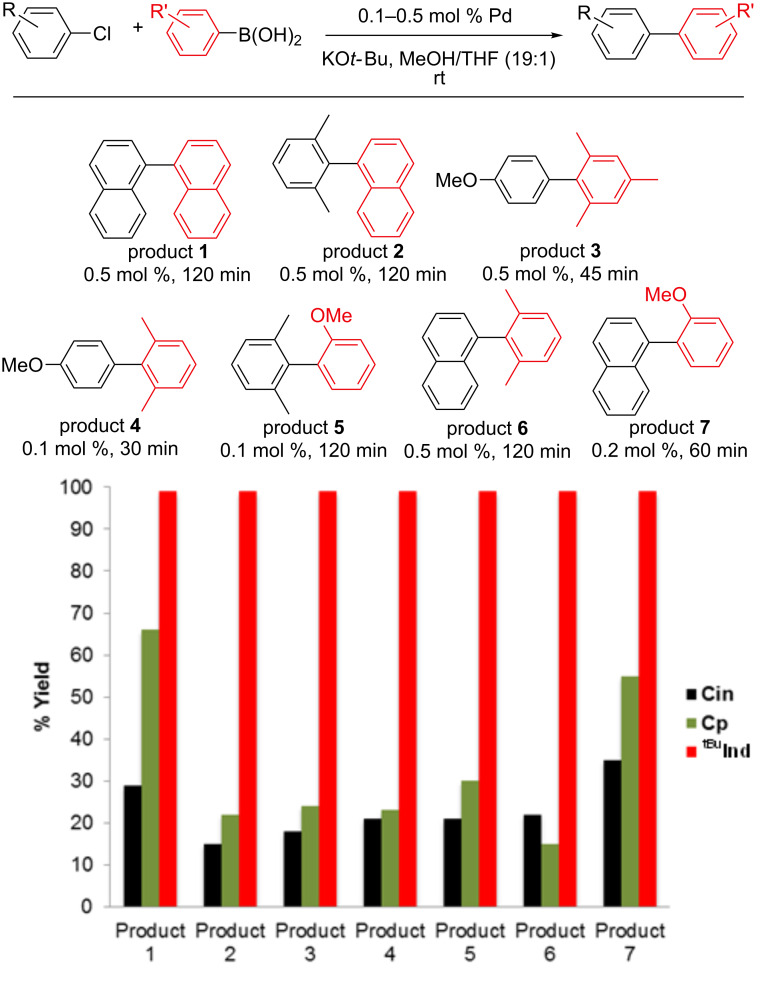
Comparison of catalytic performance of **Cin**, **Cp** and **^tBu^Ind** for a series of Suzuki–Miyaura reactions using KO*t*-Bu as the base. Yields for **Cin** and **^tBu^Ind** are from previous literature results [15]. All yields were determined using GC and are the average of two runs.

**Figure 3:**
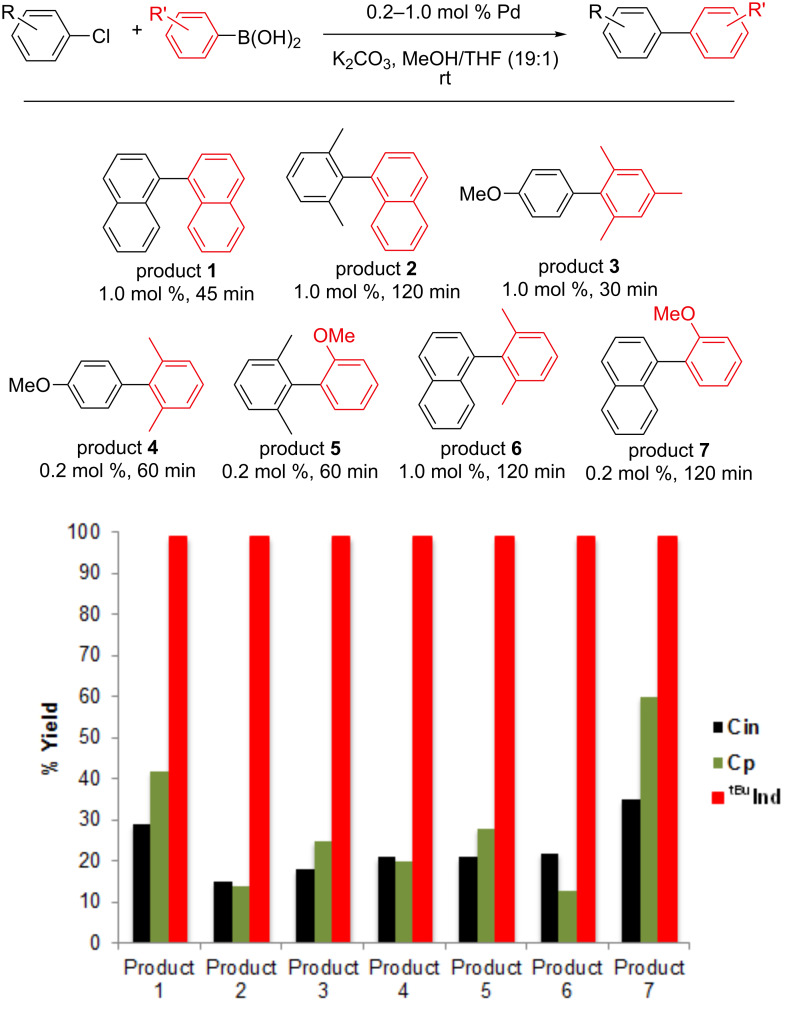
Comparison of catalytic performance of **Cin**, **Cp** and **^tBu^Ind** for a series of Suzuki–Miyaura reactions using K_2_CO_3_ as the base. Yields for **Cin** and **^tBu^Ind** are from previous literature results [15]. All yields were determined using GC and are the average of two runs.

The catalytic activity of **Cp** for Suzuki–Miyaura reactions with different substrates under both strong (KO*t*-Bu) and weak (K_2_CO_3_) base conditions is compared to **Cin** and **^tBu^Ind** in Figure 2 and Figure 3. In general, the performance of **Cp** is slightly better than **Cin**, but considerably worse than **^tBu^Ind**. At times when reactions using the **^tBu^Ind** precatalyst are complete, between 10 and 60% conversion is achieved with **Cp**. If reactions catalyzed by **Cp** are left for longer periods of time complete conversion occurs, indicating that the difference in rates is not related to rapid catalyst decomposition in the case of **Cp**. These results confirm the previously reported high activity of Cp supported precatalysts [18]. Although the difference in performance between **Cin**, **Cp** and **^tBu^Ind** varies depending on the specific substrate and catalyst loading, we are not able to discern any general trends in the data. For example, in some cases **Cp** gives better activity than **Cin** for the synthesis of di*-ortho*-substituted biaryls (products 1 and 7), whereas in another case **Cp** gives only slightly better activity (product 3). However, in general, the relative catalytic performance of the different precatalysts does not vary when the base is changed.

### Understanding the relative activity of (η^5^-Cp)Pd(IPr)Cl (Cp)

In order to understand the relative activity of **Cp** in comparison to **Cin** and **^tBu^Ind**, we measured both the rate at which it is activated to monoligated Pd(0) and its tendency to undergo comproportionation to a Pd(I) dimer. The rate of activation was measured using the same procedure that we have previously used for **Cin** and **^tBu^Ind** [16]. **Cp** was treated with base in the presence of ten equivalents of 1,3-divinyl-1,1,3,3-tetramethyldisiloxane (dvds) under a variety of conditions which are relevant to the Suzuki–Miyaura coupling and the reaction followed using ^1^H NMR spectroscopy (Table 1). The metal containing product of this reaction is the Pd(0) complex (IPr)Pd(dvds) [22]. The rate of formation of (IPr)Pd(dvds) can be used as a model for the rate of Pd(0) formation in catalysis. In all cases **Cp** is activated slower than **^tBu^Ind** [16], consistent with its inferior catalytic performance. For example, under the reaction conditions used in Table 1, entry 4, the rate of activation for **^tBu^Ind** is 7.6 ± 0.1 × 10^−4^ s^−1^ compared to 3.4 ± 0.1 × 10^−4^ s^−1^ for **Cp** [16]. In contrast, **Cp** is generally activated faster than **Cin**. The rate of activation for **Cin** under the conditions used in Table 1, entry 3 is 4.2 ± 0.1 × 10^−4^, less than half the rate of that observed for **Cp**. The conditions used in Table 1, entry 4 are the most relevant to the catalysis described above, but although in this case it appears that **Cp** is activated faster than **Cin** (3.4 ± 0.1 × 10^−4^ s^−1^ vs 1.4 ± 0.2 × 10^−4^ s^−1^), the relatively large error associated with these numbers makes a firm conclusion difficult.

**Table 1:** Rates of activation of **Cp** under different conditions in the presence of dvds.^a^

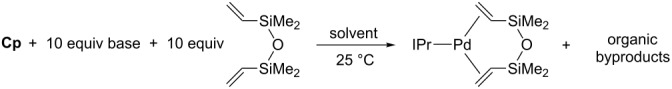				

Entry	Base	Solvent	PhB(OH)_2_ present	Rate of activation *k*_obs_ (s^−1^)^b^

1	KO*t*-Bu	iPrOH*-d*_8_^c^	No	2.8 ± 0.1 × 10^−3^
2	KO*t*-Bu	MeOH*-d*_4_	No	1.1 ± 0.1 × 10^−3^
3	K_2_CO_3_	MeOH*-d*_4_^d^	No	9.2 ± 0.2 × 10^−4^
4	K_2_CO_3_	MeOH*-d*_4_^d^	Yes^e^	3.4 ± 0.1 × 10^−4^

^a^Reaction conditions: 0.0087 mmol **Cp**, 0.087 mmol of base, 0.087 mmol of dvds in 500 μL of solvent. ^b^All rates are the average of at least two runs and were measured using ^1^H NMR spectroscopy. ^c^100 μL of THF-*d*_8_ was added along with only 400 μL of iPrOH. ^d^Two equivalents of 18-crown-6 (relative to K_2_CO_3_) were added to solubilize the K_2_CO_3_. ^e^0.0087 mmol precatalyst, 0.087 mmol phenylboronic acid, 0.096 mmol base, 0.087 mmol dvds in 500 μL MeOH*-d*_4_.

**Scheme 2:**
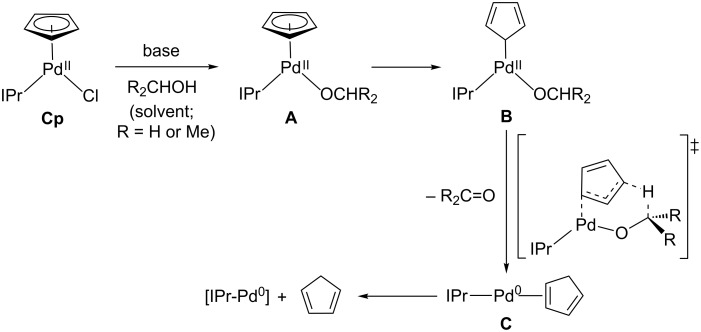
Proposed mechanism for the activation of **Cp** to monoligated Pd(0).

The mechanism of activation of **Cp** appears to be analogous to that previously described for **Cin** and **^tBu^Ind** as the organic byproducts of **Cp** activation, cyclopentadiene and either acetone (in the case of reactions performed in iPrOH) or formaldehyde (in the case of reaction performed in MeOH), are consistent with the previously reported pathway (Scheme 2) [16]. In this mechanism initial substitution of a Cl^−^ ligand in **Cp** by the solvent gives rise to the alkoxide complex **A**. Subsequently, the η^5^-Cp ring can undergo slippage to form complex **B**, with an η^1^-Cp ligand. The η^1^-Cp ligand is nucleophilic and can abstract a β-hydrogen from the alkoxide ligand to generate a Pd(0) species with a coordinated cyclopentadiene ligand (**C**). In this step the formaldehyde or acetone byproduct originating from the solvent is released. Finally, dissociation of the olefin ligand from **C** generates the active monoligated Pd(0) species, which in catalysis undergoes oxidative addition with the aryl halide, but in the case of our activation experiments is trapped by dvds.

**Scheme 3:**
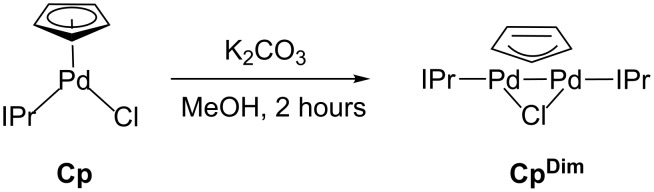
Synthesis of **Cp^Dim^**.

The considerably faster rate of activation for **Cp** compared to **Cin**, suggests that **Cp** should be a much better precatalyst than **Cin**, which is inconsistent with our catalytic results (Figure 2 and Figure 3). In our model for precatalyst performance, catalytic activity is also related to the ease at which the starting precatalyst undergoes comproportionation with monoligated Pd(0) to form a Pd(I) dimer [13]. The reaction of **Cp** with a weak base, K_2_CO_3_, in an alcohol solvent (MeOH) provided the dimeric complex, (μ-Cp)(μ-Cl)Pd_2_(IPr)_2_ (**Cp^Dim^**), in excellent yield (82%, Scheme 3). This is the same procedure we previously described for the preparation of Pd(I) dimers with a bridging chloride ligand and one bridging allyl or indenyl ligand [13].

**Figure 4:**
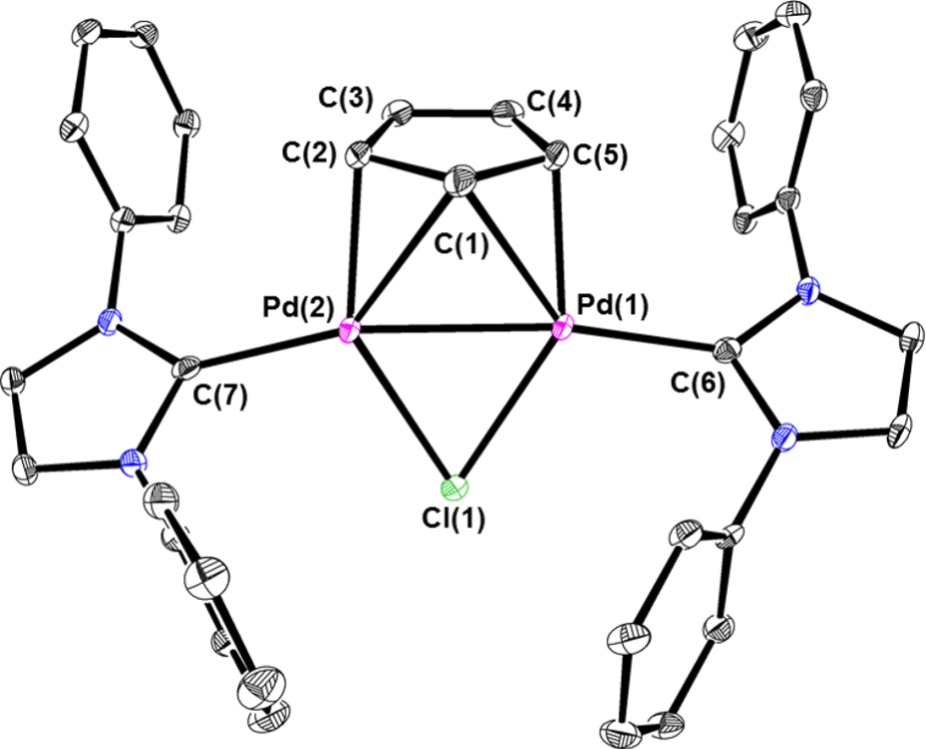
ORTEP of **Cp^Dim^** at 30% probability. Hydrogen atoms and isopropyl groups of IPr are omitted for clarity. Selected bond lengths (Å) and angles (˚) for: Pd(1)–Pd(2) 2.5669(4), Pd(1)–C(5) 2.102(4), Pd(1)–C(1) 2.480(4), Pd(2)–C(2) 2.119(4), Pd(2)–C(1) 2.449(4), Pd(1)–C(6) 2.022(4), Pd(2)–C(7) 2.031(4), Pd(1)–Cl(1) 2.402(2), Pd(2)–Cl(1) 2.398(1), C(1)–C(2) 1.433(6), C(2)–C(3) 1.463(8), C(3)–C(4) 1.347(6), C(4)–C(5) 1.465(7), C(1)–C(5) 1.438(7), Pd(1)–Cl(1)–Pd(2) 64.65(3), Pd(1)–C(1)–Pd(2) 62.8(2), Pd(1)–Pd(2)–C(7) 164.9(1), Pd(2)–Pd(1)–C(6) 171.1(1).

**Cp^Dim^** was characterized by NMR spectroscopy and X-ray crystallography (see Figure 4). The binding of the bridging Cp ligand is similar to that observed in other Pd(I) dimers supported by a bridging Cp or indenyl ligand [23–39]. The two Pd centers are bound to three carbon atoms of the bridging Cp ligand. Two of the three carbon atoms are bound to only one Pd center, while the central carbon atom binds to both Pd centers. Pd–C bond distances of almost 3 Å clearly indicate that there is no interaction between the Pd centers and the other two carbon atoms of the bridging Cp ligand. Consistent with this pseudo η^3^-binding, the C–C bond distances relating to two long bonds, two bonds of intermediate length and one short bond in the bridging Cp ligand are similar to those observed in monomeric η^3^-systems [40]. Strong evidence for a Pd–Pd single bond is provided by the Pd–Pd distance of 2.5669(4) Å [41]. Presumably for steric reasons the NHC ligands are bent away from the bridging Cp ligand and the C–Pd–Pd (C of IPr) bond angles are significantly less than 180° (Pd(1)–Pd(2)–C(7) 164.9(1) and Pd(2)–Pd(1)–C(6) 171.1(1)).

**Scheme 4:**
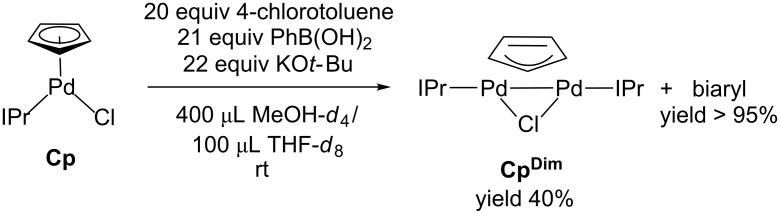
Observation of **Cp^Dim^** under modified catalytic conditions.

**Scheme 5:**
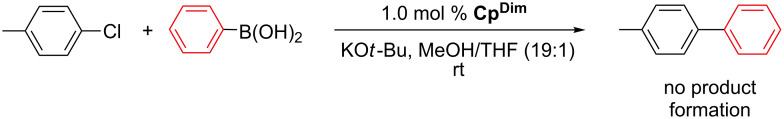
**Cp^Dim^** is not an active precatalyst for a Suzuki–Miyaura reaction at room temperature.

To determine if **Cp^Dim^** is catalytically relevant modified conditions were used to allow for the reaction to be monitored by ^1^H NMR spectroscopy (Scheme 4). In order to observe the Pd containing species, an increased catalyst loading was used, 4 mol % **Cp**, compared to the loadings described in Figure 2 and Figure 3. Peaks consistent with the formation of **Cp^Dim^** are observed during catalysis, and approximately 40% of the Pd is in the form of **Cp^Dim^** upon completion of the catalytic reaction. In contrast, for **Cin** under the same conditions, only a small amount of Pd was determined to be in the form of a Pd(I) dimer [13]. This suggests that **Cp** is more likely to undergo dimerization than **Cin**. **Cp^Dim^** was confirmed to be a poor catalyst under the conditions employed in Figure 2 (Scheme 5). This result is indicative of **Cp^Dim^** as an off-cycle deactivation product, which reduces the amount of the active Pd(0) species in solution.

**Scheme 6:**
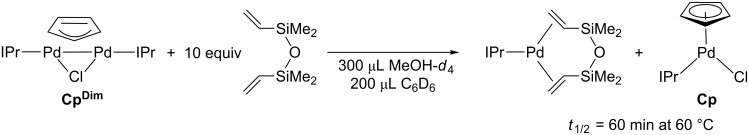
Disproportionation of **Cp^Dim^** with dvds.

Previously, we have demonstrated that the comproportionation of Pd(0) and Pd(II) species to IPr supported Pd(I) dimers with one bridging allyl and one bridging chloride ligand is reversible [13–14]. One method to measure the rate of disproportionation of Pd(I) dimers is to react these species with a trapping agent for Pd(0), such as dvds. This results in the formation of the Pd(0) species (IPr)Pd(dvds) and a Pd(II) species of the form (η^3^-allyl)Pd(IPr)(Cl). We examined the tendency of **Cp^Dim^** to undergo disproportionation in the presence of dvds. The disproportionation of **Cp^Dim^** is extremely difficult and at 60 °C the half-life for the formation of **Cp** and (IPr)Pd(dvds) is 60 minutes (Scheme 6). In contrast, in the presence of dvds (μ-cinnamyl)(μ-Cl)Pd_2_(IPr)_2_ undergoes full disproportionation in approximately 40 minutes at 40 °C, while for (μ-allyl)(μ-Cl)Pd_2_(IPr)_2_ the reaction is complete in less than 10 minutes at room temperature [13]. Although these results show that disproportionation of **Cp^Dim^** is more difficult than related allyl species, they provide no information on whether this is related to thermodynamic or kinetic effects.

**Scheme 7:**
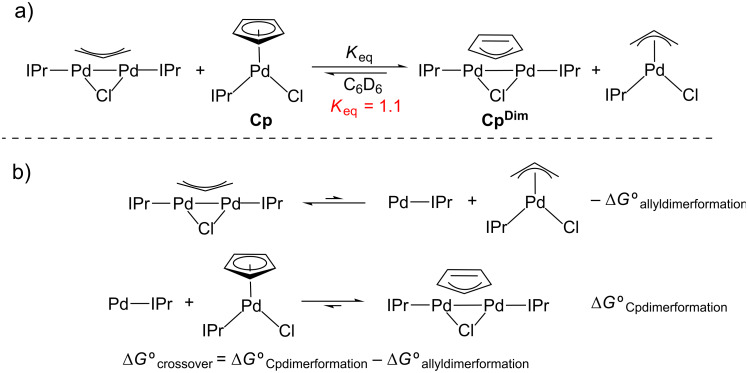
a) Crossover experiment between **Cp** and (μ-allyl)(μ-Cl)Pd_2_(IPr)_2_. b) Crossover experiment expressed as the sum of disproportionation and comproportionation half reactions.

To probe the relative thermodynamic favorability of dimer formation between allyl and Cp systems we performed a crossover experiment (Scheme 7a). In this experiment (μ-allyl)(μ-Cl)Pd_2_(IPr)_2_ was mixed with **Cp**. The products of crossover are **Cp^Dim^** and (η^3^-allyl)Pd(IPr)(Cl) and our experiments indicate that the equilibrium favors these species. The crossover reaction can be described as the combination of the disproportionation of the allyl dimer and the comproportionation of **Cp** with IPr-Pd(0) (Scheme 7b). From these results, we conclude that the comproportionation reaction to form **Cp^Dim^** is more exergonic than in the allyl case (**|**ΔG°_Cpdimerformation_**|** > **|**ΔG°_allyldimerformation_**|** in Scheme 7b). The results of this experiment indicate that in part disproportionation of **Cp^Dim^** to form **Cp** and L-Pd(0) is more challenging than the corresponding allyl dimer for thermodynamic reasons.

## Conclusion

We have performed the first detailed comparative investigation of the catalytic activity for the Suzuki–Miyaura reaction of (η^5^-Cp)Pd(IPr)(Cl) (**Cp**), with the related commercially available catalysts (η^3^-cinnamyl)Pd(IPr)(Cl) (**Cin**) and (η^3^-1-*t*-Bu-indenyl)Pd(IPr)(Cl) (**^tBu^Ind**). We found that **Cp** is a slightly more efficient catalyst than **Cin**, but significantly less active than **^tBu^Ind**. The low activity of **Cp** in comparison to **^tBu^Ind** is related both to its slower rate of activation to the monoligated Pd(0) active species and its tendency to form a significant amount of the inactive Pd(I) dimer (μ-Cp)(μ-Cl)Pd_2_(IPr)_2_ (**Cp^Dim^**) under catalytic conditions. The formation of this inactive dimer also explains why **Cp** is only a slightly more active precatalyst than **Cin**, which activates slower than **Cp**, but is less likely to form the corresponding inactive Pd(I) dimer. In principle, the addition of steric bulk to **Cp** could prevent the formation of a Pd(I) dimer and result in a more active precatalyst. However, an additional challenge that must be overcome if practical precatalyst scaffolds based on a Cp ligand are to be developed is that the synthetic routes to these species are currently not amenable to rapid ligand screening in an analogous fashion to **Cin** and **^tBu^Ind**.

## Experimental

### General methods

As previously described in [13] and [15], experiments were performed under a dinitrogen atmosphere in an M-Braun dry box or using standard Schlenk techniques unless otherwise stated. Under standard glovebox conditions, purging was not performed between uses of pentane, benzene and toluene; thus when any of these solvents were used, traces of all these solvents were in the atmosphere and could be found intermixed in the solvent bottles. Stainless steel cannulas were used to transfer moisture- and air-sensitive liquids on a Schlenk line or in a dry box. THF, diethyl ether, and toluene were dried by passage through a column of activated alumina followed by storage under dinitrogen. All commercial chemicals were used as received; exceptions where noted. MeOH (J. T. Baker) and iPrOH (Macron Fine Chemicals) were not dried but were degassed by sparging with dinitrogen for one hour and stored under dinitrogen. Potassium *tert*-butoxide (99.99%, sublimed) was purchased from Aldrich. Potassium carbonate was purchased from Mallinckrodt and ground up with a mortar and pestle and stored in an oven at 130 °C prior to use. 1,3-Divinyltetramethyldisiloxane was purchased from TCI. Deuterated solvents were obtained from Cambridge Isotope Laboratories. MeOH-*d*_4_ and THF-*d*_8_ were not dried but were degassed prior to use through three freeze-pump-thaw cycles. Agilent-400, -500 and -600 spectrometers were used to record NMR spectra at ambient probe temperatures. Gas chromatography analyses (GC) were performed on a Shimadzu GC-2010 Plus apparatus equipped with a flame ionization detector and a Shimadzu SHRXI-5MS column (30 m, 250 μm inner diameter, film: 0.25 μm). The following conditions were utilized for GC analyses: flow rate 1.23 mL/min constant flow, column temperature 50 °C (held for 5 min), 20 °C/min increase to 300 °C (held for 5 min), total time 22.5 min. Literature procedures were used to prepare the following compounds: (η^3^-cinnamyl)Pd(IPr)(Cl) (**Cin**) [11], (η^3^-1-*t*-Bu-indenyl)Pd(IPr)(Cl) (**^tBu^Ind**) [15], (η^5^-Cp)Pd(IPr)(Cl) (**Cp**) [18] (μ-allyl)(μ-Cl)Pd_2_(IPr)_2_ [13].

### X-ray crystallography

X-ray diffraction experiments were carried out on a Rigaku MicroMax-007HF diffractometer coupled to a Saturn994+ CCD detector with Cu Kα radiation (λ = 1.54178 Å) at −180 °C. The crystals were mounted on MiTeGen polyimide loops with immersion oil. The data frames were processed using Rigaku CrystalClear and corrected for Lorentz and polarization effects. Using Olex2 [42] the structure was solved with the XS [43] structure solution program by Patterson methods and refined with the XL [43] refinement package using least-squares minimization. The non-hydrogen atoms were refined anisotropically. Hydrogen atoms were refined using the riding model unless otherwise stated.

### Synthetic procedures and characterizing data

#### (μ-Cp)(μ-Cl)Pd_2_(IPr)_2_ (**Cp^Dim^**)

(η^5^-Cp)Pd(IPr)(Cl) (**Cp**) (0.250 g, 0.42 mmol) and K_2_CO_3_ (0.116 g, 0.84 mmol) were added to a 100 mL Schlenk flask. Degassed MeOH (30 mL) was added to the flask via cannula. The reaction mixture was stirred at room temperature for 2 hours. The precipitate was filtered in air and washed with water to remove excess salts. The solid was washed with pentane and dried under vacuum to give **Cp^Dim^** as a red solid. Yield: 0.188 g, 82%. X-ray quality crystals were grown from a saturated toluene solution layered with pentane (V(toluene):V(pentane) = 1:2) at −35 °C. ^1^H NMR (C_6_D_6_, 400 MHz) 7.18 (t, *J* = 7.7 Hz, 4H), 7.11 (d, *J* = 7.7 Hz, 8H), 6.62 (s, 4H), 4.39 (s, 5H), 3.13 (sept, *J* = 6.8 Hz, 8H), 1.35 (d, *J* = 6.9 Hz, 24H), 1.11 (d, *J* = 6.9 Hz, 24H); ^13^C{^1^H} NMR (C_6_D_6_, 100 MHz) 186.5, 146.0, 137.3, 128.9, 123.4, 122.2, 84.3, 28.5, 25.3, 23.1.

### Representative procedures for catalytic Suzuki–Miyaura reactions with **Cp**

#### KO*t*-Bu conditions

Reactions were performed under dinitrogen in a 1 dram vial containing a flea stir bar and sealed with a septum cap. To the vial was added 950 μL of a MeOH stock solution, containing 0.5263 M aryl chloride, 0.5525 M boronic acid, 0.5789 M KO*t*-Bu and 0.2632 M naphthalene. The vial was then heated using an aluminum block heater set to 25 °C. After thermal equilibration, the reaction was initiated via the addition of 50 μL of the appropriate precatalyst solution in THF (0.1 M [Pd]). Aliquots (≈50–100 μL) were removed at reaction times indicated. The aliquots were purified by filtration through pipet filters containing approximately 1 cm of silica and eluted with 1–1.2 mL of ethyl acetate directly into GC vials. Conversion was determined by comparison of the GC responses of product and the internal naphthalene standard. Biaryl products were initially synthesized using literature procedures [15], identified using NMR spectroscopy by comparison to the literature chemical shifts [11] and then these pure samples used to generate calibration plots for the GC.

#### K_2_CO_3_ conditions

Potassium carbonate (0.75 mmol) was transferred on the benchtop into a 1 dram vial containing a flea stir bar. The vial was sealed with a septum cap, and placed under dinitrogen (by cycling three times between vacuum and dinitrogen) on a Schlenk line through a needle. To the vial was added 950 μL of a MeOH stock solution, containing 0.5263 M aryl chloride, 0.5525 M boronic acid and 0.2632 M naphthalene. The vial was then heated using an aluminum block heater set to 25 °C. After thermal equilibration, the reaction was initiated via the addition of 50 μL of the appropriate precatalyst solution in THF (0.1 M [Pd]). Aliquots (≈50–100 μL) were removed at reaction times indicated. The aliquots were purified by filtration through pipet filters containing approximately 1 cm of silica and eluted with 1–1.2 mL of ethyl acetate directly into GC vials. Conversion was determined by comparison of the GC responses of product and the internal naphthalene standard. Biaryl products were initially synthesized using literature procedures [15], identified using NMR spectroscopy by comparison to the literature chemical shifts [11] and then these pure samples used to generate calibration plots for the GC.

### Experiments on activation of Pd(II) to Pd(0)

#### Experimental details for Table 1: Rates of activation of **Cp** under different conditions in the presence of dvds

**iPrOH-*d*_8_/KO*t*-Bu experiments:** KO*t*-Bu (9.8 mg, 0.087 mmol) was dissolved in 300 μL of iPrOH-*d*_8_ along with 100 μL of a 0.87 M solution of dvds in iPrOH*-d*_8_. **Cp** (5.2 mg, 0.0087 mmol) was dissolved in 100 μL of THF-*d*_8_. These solutions were combined in a J. Young NMR tube at −78 °C. The reaction mixture was degassed on a Schlenk line, after which dinitrogen was introduced into the NMR tube. An array of ^1^H NMR spectra was taken at 25 °C over the course of 3 hours. During this time, the growth of the methyl protons of the (IPr)Pd(dvds) [22] product were monitored.

**MeOH-*d*_4_/KO*t*-Bu experiments:** KO*t*-Bu (9.8 mg, 0.087 mmol) was dissolved in 300 μL of MeOH-*d*_4_ along with 100 μL of a 0.87 M solution of dvds in MeOH-*d*_4_. **Cp** (5.2 mg, 0.0087 mmol) was dissolved in 100 μL of MeOH-*d*_4_. These solutions were combined in a J. Young NMR tube at −78 °C. The reaction mixture was degassed on a Schlenk line, after which dinitrogen was introduced into the NMR tube. An array of ^1^H NMR spectra was taken at 25 °C over the course of 3 hours. During this time, the growth of the methyl protons of the (IPr)Pd(dvds) [22] product were monitored.

**MeOH-*d*_4_/K_2_CO_3_ experiments:** K_2_CO_3_ (12.0 mg, 0.087 mmol) and 18-crown-6 ether (46.0 mg, 0.174 mmol) were dissolved in 300 μL of MeOH-*d*_4_ along with 100 μL of a 0.87 M solution of dvds in MeOH-*d*_4_. **Cp** (5.2 mg, 0.0087 mmol) was dissolved in 100 μL of MeOH-*d*_4_. These solutions were combined in a J. Young NMR tube at −78 °C. The reaction mixture was degassed on a Schlenk line, after which dinitrogen was introduced into the NMR tube. An array of ^1^H NMR spectra was taken at 25 °C over the course of 3 hours. The strong –CH_2_ peak from the 18-crown-6 ether was suppressed by presaturating its signal during the experiment. During this time, the growth of the methyl protons of the (IPr)Pd(dvds) [22] product were monitored.

**MeOH-*d*_4_/K_2_CO_3_/PhB(OH)_2_ experiments:** KO*t*-Bu (10.8 mg, 0.096 mmol) and phenylboronic acid (10.6 mg, 0.087 mmol) were dissolved in 300 μL of MeOH-*d*_4_ along with 100 μL of a 0.87 M solution of dvds in MeOH-*d*_4_. **Cp** (5.2 mg, 0.0087 mmol) was dissolved in 100 μL of MeOH-*d*_4_. These solutions were combined in a J. Young NMR tube at –78 °C. The reaction mixture was degassed on a Schlenk line, after which dinitrogen was introduced into the NMR tube. An array of ^1^H NMR spectra was taken at 25 °C over the course of 3 hours. During this time, the growth of the methyl protons of the (IPr)Pd(dvds) [22] product were monitored.

**Catalysis using Cp under NMR conditions:** In a glovebox, phenylboronic acid (10.0 mg, 0.082 mmol), 4-chlorotoluene (9.2 μL, 0.0781 mmol), KO*t*-Bu (9.6 mg, 0.0859 mmol) and 2,6-dimethoxytoluene (6.0 mg, 0.039 mmol) were dissolved in 400 μL of MeOH-*d*_4_. **Cp** (1.8 mg, 0.0031 mmol) was dissolved in 100 μL of THF*-d*_8_. These solutions were combined in a J. Young NMR tube and the reaction was monitored by ^1^H NMR spectroscopy for one hour at 25 °C. After this time, the solvent mixture was removed on a Schlenk line and benzene-*d*_6_ was added. A final ^1^H NMR spectrum was recorded to identify the Pd containing products of the reaction. **Cp^Dim^** was observed as the main Pd containing product, with a yield of 40% compared to the internal standard 2,6-dimethoxytoluene.

**Catalysis using Cp^Dim^ as precatalyst:** 0.05 mmol of **Cp^Dim^** was transferred into a 1 mL volumetric flask in a glovebox. The precatalyst was dissolved in THF, and the solution was diluted to 1 mL. The solution was transferred to a flask with a Kontes valve. Reactions were performed under dinitrogen in a 1 dram vial containing a flea stir bar and sealed with a septum cap. To the vial was added 950 μL of the MeOH stock solution described above. The vial was then heated using an aluminum block heater set to 25 °C. After thermal equilibration, the reaction was initiated via the addition of 50 μL of the THF solution containing **Cp^Dim^** (0.1 M [Pd]). Aliquots (≈50–100 μL) were removed at 30 and 60 minutes. The aliquots were purified by filtration through pipet filters containing approximately 1 cm of silica and eluted with 1–1.2 mL of ethyl acetate directly into GC vials. Conversion was determined by comparison of the GC responses of product and the internal naphthalene standard. No conversion to the biphenyl product was observed at either time point.

**Disproportionation of Cp^Dim^ using dvds:** In a nitrogen filled glovebox, **Cp^Dim^** (5.5 mg, 0.005 mmol), dvds (9.2 mg, 0.05 mmol) and 2,6-dimethocytoluene (0.8 mg, 0.005 mmol) were added to a vial. MeOH-*d*_4_ (300 μL) and deuterated benzene (200 μL) were added and the homogeneous mixture was transferred to a J. Young tube and sealed. The contents were heated at 60 °C for one hour, at which time an NMR spectrum was recorded. The methyl protons of the product (IPr)Pd(dvds) [22] were compared to the internal standard. At one hour, the reaction had reached 50% conversion.

**Crossover experiment using Cp and (μ-allyl)(μ-Cl)Pd_2_(IPr)_2_:** In a nitrogen-filled glovebox, (μ-allyl)(μ-Cl)Pd_2_(IPr)_2_ (4.0 mg, 0.00375 mmol) and **Cp** (2.2 mg, 0.00375 mmol) were added to a vial. C_6_D_6_ (0.5 mL) was added and the solution was transferred to a J. Young tube and sealed. The mixture was heated to 60 °C and allowed to equilibrate over 36 hours. At this time, an NMR spectrum was recorded at room temperature. The equilibrium constant was calculated by using relative integrations of the Cp protons from **Cp** and **Cp^Dim^** to yield a *K* of 1.1.

## Supporting Information

File 1 ^1^H NMR spectrum for **^tBu^Ind**, **Cp** and **^Cp^Dim** and crystallographic information for **^Cp^Dim**.

File 2 Crystallographic information file for **^Cp^Dim**.
