# Supplementary material for: Comparison of the catalytic activity for the Suzuki–Miyaura reaction of (η5-Cp)Pd(IPr)Cl with (η3-cinnamyl)Pd(IPr)(Cl) and (η3-1-t-Bu-indenyl)Pd(IPr)(Cl)
Source: Beilstein J Org Chem. 2015 Dec 8;11:2476–86. doi: 10.3762/bjoc.11.269 (PMC4685905; doi:10.3762/bjoc.11.269)
Supplement: File 1 — 1H NMR spectrum for tBuInd, Cp and CpDim and crystallographic information for CpDim. [file Beilstein_J_Org_Chem-11-2476-s001.pdf]

Supporting Information

for

Comparison of the catalytic activity for the Suzuki–Miyaura reaction of (η^5-Cp)Pd(IPr)Cl with (η^3-cinnamyl)Pd(IPr)(Cl) and (η^3-1-*t*-Bu-indenyl)Pd(IPr)(Cl)

Patrick R. Melvin, Nilay Hazari*, Hannah M. C. Lant, Ian L. Peczak and Hemali P. Shah

Address: The Department of Chemistry, Yale University, P. O. Box 208107, New Haven, Connecticut, 06520, USA.

Email: Nilay Hazari - nilay.hazari@yale.edu.

*Corresponding author

^{1}H NMR spectrum for ^{tBu}Ind, Cp and ^{Cp}Dim and crystallographic information for ^{Cp}Dim

Table of Contents:

^{1}H NMR Spectrum of ^{tBu}**Ind** S2

^{1}H NMR Spectrum of **Cp** S3

^{1}H NMR Spectrum of **Cp**^{Dim} S3

Crystallographic Information for **Cp**^{Dim} S4

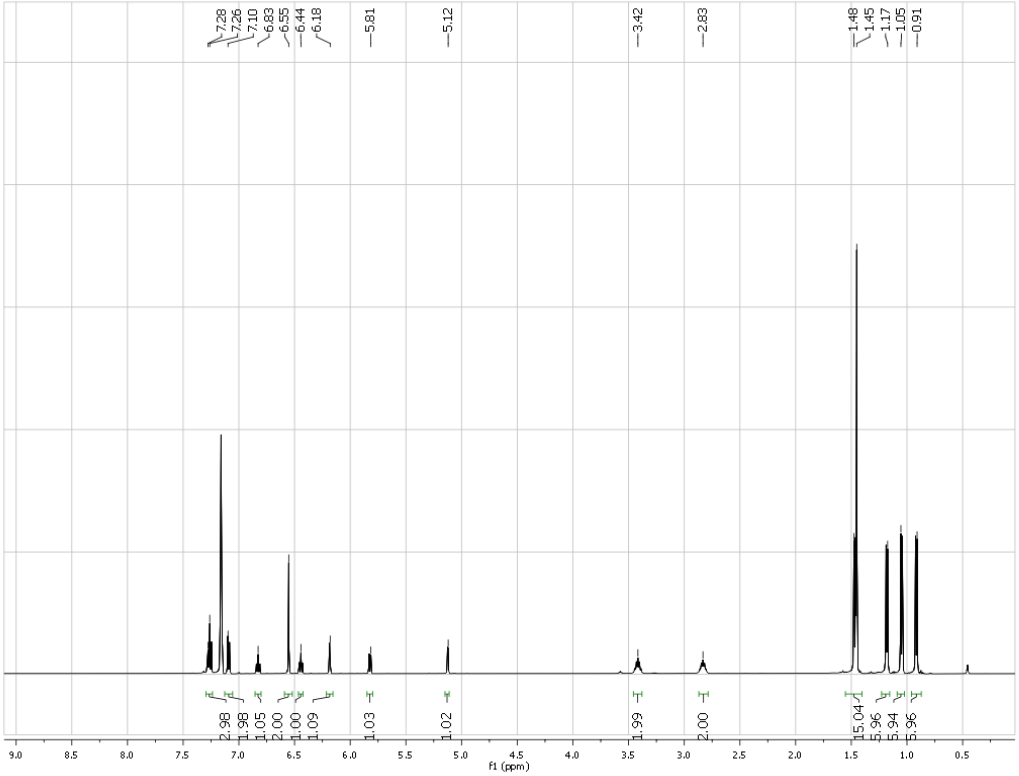

Figure S2: ^{1}H NMR spectrum of **^{tBu}Ind**.

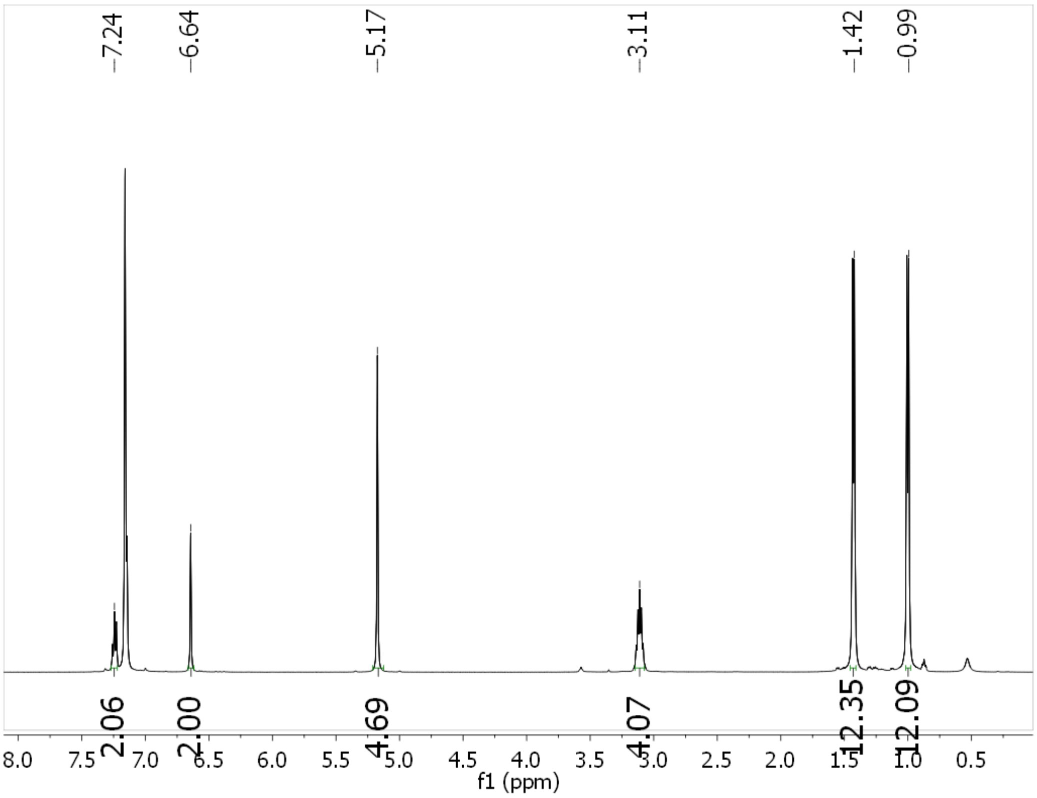

Figure S3: ^{1}H NMR spectrum of **Cp**.

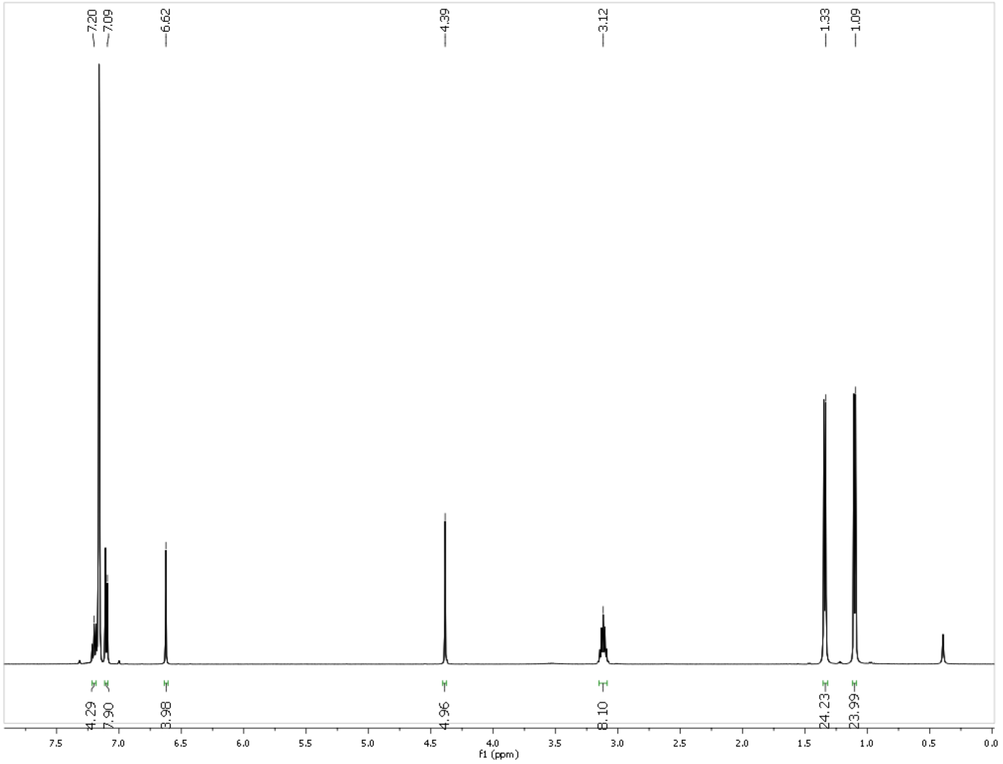

Figure S4: ^{1}H NMR spectrum of **Cp^{Dim}**.

Table S1: Crystal data and structure refinement for $\mathbf{Cp^{Dim}}$.

Empirical formula	$C_{59}H_{77}ClN_4Pd_2$	
Formula weight	1090.49	
Temperature	93(2) K	
Wavelength	1.54178 Å	
Crystal system	Monoclinic	
Space group	$P\ 2_1$	
Unit cell dimensions	a = 11.5881(2) Å	α = 90°.
	b = 12.3150(2) Å	β = 100.473(7)°.
	c = 19.3155(14) Å	γ = 90°.
Volume	2710.5(2) $Å^3$	
Z	2	
Density (calculated)	1.336 Mg/m^3	
Absorption coefficient	6.101 mm^{-1}	
F(000)	1136	
Crystal size	0.100 x 0.050 x 0.050 mm^3	
Crystal color and habit	Red Block	
Diffractometer	Rigaku Saturn 944+ CCD	
Theta range for data collection	2.326 to 63.677°.	
Index ranges	$-13 \leq h \leq 13$, $-13 \leq k \leq 14$, $-22 \leq l \leq 22$	
Reflections collected	90929	
Independent reflections	8645 [R(int) = 0.0821]	
Observed reflections (I > 2sigma(I))	8627	
Completeness to theta = 63.677°	99.6 %	
Absorption correction	Semi-empirical from equivalents	
Max. and min. transmission	0.750 and 0.328	
Solution method	SHELXT-2014/5 (Sheldrick, 2014)	
Refinement method	SHELXL-2014/7 (Sheldrick, 2014)	
Data / restraints / parameters	8645 / 1 / 621	
Goodness-of-fit on F^2	1.055	
Final R indices [I>2sigma(I)]	R1 = 0.0218, wR2 = 0.0555	
R indices (all data)	R1 = 0.0218, wR2 = 0.0556	
Absolute structure parameter	0.454(5)	
Largest diff. peak and hole	0.554 and -0.393 e.$Å^{-3}$	

Table S2: Atomic coordinates (× 10^4) and equivalent isotropic displacement parameters (Å^2× 10^3) for **Cp^{Dim}**. U(eq) is defined as one third of the trace of the orthogonalized U^{ij} tensor.

	x	y	z	U(eq)
Pd(1)	2688(1)	4912(1)	3292(1)	18(1)
Pd(2)	3623(1)	5239(1)	2203(1)	18(1)
Cl(1)	1610(1)	4696(1)	2117(1)	33(1)
N(1)	1234(2)	5361(3)	4460(1)	18(1)
N(2)	792(2)	3828(3)	3952(1)	19(1)
N(3)	4834(2)	6067(3)	979(1)	17(1)
N(4)	3967(2)	4538(3)	775(1)	18(1)
C(1)	4292(3)	5437(3)	3900(2)	24(1)
C(2)	4822(3)	4890(4)	3381(2)	25(1)
C(3)	5152(3)	5672(4)	2902(2)	24(1)
C(4)	4831(3)	6734(4)	3157(2)	26(1)
C(5)	4334(3)	6594(4)	3730(2)	26(1)
C(6)	1549(3)	4688(3)	3966(2)	18(1)
C(7)	321(3)	4918(3)	4753(2)	22(1)
C(8)	53(3)	3964(3)	4436(2)	22(1)
C(9)	4160(3)	5347(3)	1265(2)	17(1)
C(10)	5045(3)	5708(3)	326(2)	21(1)
C(11)	4504(3)	4751(3)	199(2)	22(1)
C(12)	1840(3)	6335(3)	4710(2)	20(1)
C(13)	1591(3)	7298(3)	4329(2)	20(1)
C(14)	2199(3)	8229(3)	4597(2)	21(1)
C(15)	3006(3)	8208(3)	5216(2)	23(1)
C(16)	3230(3)	7243(3)	5585(2)	25(1)
C(17)	2656(3)	6283(3)	5344(2)	22(1)
C(18)	704(3)	7360(3)	3647(2)	22(1)
C(19)	-235(3)	8218(4)	3691(2)	32(1)
C(20)	1324(4)	7594(4)	3022(2)	29(1)
C(21)	2898(3)	5240(4)	5768(2)	29(1)
C(22)	2356(4)	5316(4)	6436(2)	37(1)
C(23)	4197(3)	4973(4)	5967(2)	37(1)
C(24)	911(3)	2819(3)	3591(2)	21(1)

C(25)	162(3)	2599(3)	2944(2)	25(1)
C(26)	306(4)	1616(4)	2629(2)	34(1)
C(27)	1155(4)	884(4)	2927(2)	40(1)
C(28)	1876(4)	1115(4)	3564(2)	32(1)
C(29)	1761(3)	2084(3)	3916(2)	26(1)
C(30)	-760(3)	3413(4)	2610(2)	26(1)
C(31)	-1907(3)	3293(4)	2898(2)	36(1)
C(32)	-1028(4)	3338(4)	1806(2)	31(1)
C(33)	2567(3)	2330(3)	4608(2)	26(1)
C(34)	2503(4)	1466(4)	5167(2)	42(1)
C(35)	3823(4)	2484(4)	4498(2)	40(1)
C(36)	5196(3)	7107(3)	1278(2)	18(1)
C(37)	4355(3)	7946(3)	1223(2)	20(1)
C(38)	4727(3)	8942(3)	1522(2)	26(1)
C(39)	5883(3)	9101(4)	1849(2)	28(1)
C(40)	6685(3)	8267(3)	1887(2)	26(1)
C(41)	6367(3)	7254(3)	1602(2)	23(1)
C(42)	3079(3)	7790(3)	869(2)	25(1)
C(43)	2712(3)	8620(4)	280(2)	35(1)
C(44)	2274(4)	7848(4)	1412(2)	38(1)
C(45)	7267(3)	6350(4)	1640(2)	26(1)
C(46)	7964(4)	6443(4)	1040(2)	37(1)
C(47)	8122(4)	6317(4)	2344(2)	36(1)
C(48)	3155(3)	3659(3)	801(2)	20(1)
C(49)	2026(3)	3760(3)	390(2)	23(1)
C(50)	1248(3)	2905(4)	418(2)	28(1)
C(51)	1565(4)	2012(4)	836(2)	33(1)
C(52)	2676(4)	1951(4)	1251(2)	29(1)
C(53)	3499(3)	2774(3)	1242(2)	22(1)
C(54)	1634(3)	4777(4)	-26(2)	30(1)
C(55)	730(5)	5395(5)	303(2)	55(2)
C(56)	1154(5)	4559(5)	-806(2)	47(1)
C(57)	4709(3)	2719(3)	1702(2)	24(1)
C(58)	4696(4)	2150(4)	2404(2)	32(1)
C(59)	5581(3)	2169(4)	1311(2)	32(1)

Table S3: Bond lengths [Å] and angles [°] for **Cp^{Dim}**.

Pd(1)-C(6)	2.034(3)
Pd(1)-C(1)	2.112(3)
Pd(1)-Cl(1)	2.3984(9)
Pd(1)-C(2)	2.448(3)
Pd(1)-Pd(2)	2.5669(3)
Pd(2)-C(9)	2.024(3)
Pd(2)-C(3)	2.093(3)
Pd(2)-Cl(1)	2.4028(8)
Pd(2)-C(2)	2.479(3)
N(1)-C(6)	1.363(4)
N(1)-C(7)	1.398(4)
N(1)-C(12)	1.430(5)
N(2)-C(6)	1.373(4)
N(2)-C(8)	1.388(4)
N(2)-C(24)	1.443(5)
N(3)-C(9)	1.364(5)
N(3)-C(10)	1.399(4)
N(3)-C(36)	1.435(5)
N(4)-C(9)	1.364(4)
N(4)-C(11)	1.396(4)
N(4)-C(48)	1.441(5)
C(1)-C(2)	1.435(5)
C(1)-C(5)	1.464(6)
C(1)-H(1)	0.94(4)
C(2)-C(3)	1.435(6)
C(2)-H(2)	0.92(5)
C(3)-C(4)	1.469(6)
C(3)-H(3)	0.89(4)
C(4)-C(5)	1.348(5)
C(4)-H(4)	0.9500
C(5)-H(5)	0.9500
C(7)-C(8)	1.335(6)
C(7)-H(7)	0.9500
C(8)-H(8)	0.9500

C(10)-C(11)	1.336(6)
C(10)-H(10)	0.9500
C(11)-H(11)	0.9500
C(12)-C(13)	1.398(5)
C(12)-C(17)	1.407(5)
C(13)-C(14)	1.395(5)
C(13)-C(18)	1.517(5)
C(14)-C(15)	1.377(5)
C(14)-H(14)	0.9500
C(15)-C(16)	1.385(6)
C(15)-H(15)	0.9500
C(16)-C(17)	1.394(6)
C(16)-H(16)	0.9500
C(17)-C(21)	1.521(5)
C(18)-C(19)	1.530(5)
C(18)-C(20)	1.540(5)
C(18)-H(18)	1.0000
C(19)-H(19A)	0.9800
C(19)-H(19B)	0.9800
C(19)-H(19C)	0.9800
C(20)-H(20A)	0.9800
C(20)-H(20B)	0.9800
C(20)-H(20C)	0.9800
C(21)-C(23)	1.520(5)
C(21)-C(22)	1.536(5)
C(21)-H(21)	1.0000
C(22)-H(22A)	0.9800
C(22)-H(22B)	0.9800
C(22)-H(22C)	0.9800
C(23)-H(23A)	0.9800
C(23)-H(23B)	0.9800
C(23)-H(23C)	0.9800
C(24)-C(29)	1.399(5)
C(24)-C(25)	1.413(5)
C(25)-C(26)	1.380(6)
C(25)-C(30)	1.520(6)

C(26)-C(27)	1.380(6)
C(26)-H(26)	0.9500
C(27)-C(28)	1.385(6)
C(27)-H(27)	0.9500
C(28)-C(29)	1.391(6)
C(28)-H(28)	0.9500
C(29)-C(33)	1.516(5)
C(30)-C(32)	1.529(5)
C(30)-C(31)	1.539(5)
C(30)-H(30)	1.0000
C(31)-H(31A)	0.9800
C(31)-H(31B)	0.9800
C(31)-H(31C)	0.9800
C(32)-H(32A)	0.9800
C(32)-H(32B)	0.9800
C(32)-H(32C)	0.9800
C(33)-C(35)	1.520(5)
C(33)-C(34)	1.528(6)
C(33)-H(33)	1.0000
C(34)-H(34A)	0.9800
C(34)-H(34B)	0.9800
C(34)-H(34C)	0.9800
C(35)-H(35A)	0.9800
C(35)-H(35B)	0.9800
C(35)-H(35C)	0.9800
C(36)-C(41)	1.400(5)
C(36)-C(37)	1.410(5)
C(37)-C(38)	1.390(6)
C(37)-C(42)	1.524(5)
C(38)-C(39)	1.388(5)
C(38)-H(38)	0.9500
C(39)-C(40)	1.379(6)
C(39)-H(39)	0.9500
C(40)-C(41)	1.385(6)
C(40)-H(40)	0.9500
C(41)-C(45)	1.518(5)

C(42)-C(44)	1.527(5)
C(42)-C(43)	1.531(6)
C(42)-H(42)	1.0000
C(43)-H(43A)	0.9800
C(43)-H(43B)	0.9800
C(43)-H(43C)	0.9800
C(44)-H(44A)	0.9800
C(44)-H(44B)	0.9800
C(44)-H(44C)	0.9800
C(45)-C(47)	1.530(5)
C(45)-C(46)	1.533(5)
C(45)-H(45)	1.0000
C(46)-H(46A)	0.9800
C(46)-H(46B)	0.9800
C(46)-H(46C)	0.9800
C(47)-H(47A)	0.9800
C(47)-H(47B)	0.9800
C(47)-H(47C)	0.9800
C(48)-C(53)	1.396(5)
C(48)-C(49)	1.408(5)
C(49)-C(50)	1.393(6)
C(49)-C(54)	1.512(6)
C(50)-C(51)	1.374(6)
C(50)-H(50)	0.9500
C(51)-C(52)	1.388(6)
C(51)-H(51)	0.9500
C(52)-C(53)	1.394(5)
C(52)-H(52)	0.9500
C(53)-C(57)	1.518(5)
C(54)-C(55)	1.525(6)
C(54)-C(56)	1.531(5)
C(54)-H(54)	1.0000
C(55)-H(55A)	0.9800
C(55)-H(55B)	0.9800
C(55)-H(55C)	0.9800
C(56)-H(56A)	0.9800

C(56)-H(56B)	0.9800
C(56)-H(56C)	0.9800
C(57)-C(59)	1.525(6)
C(57)-C(58)	1.529(5)
C(57)-H(57)	1.0000
C(58)-H(58A)	0.9800
C(58)-H(58B)	0.9800
C(58)-H(58C)	0.9800
C(59)-H(59A)	0.9800
C(59)-H(59B)	0.9800
C(59)-H(59C)	0.9800
C(6)-Pd(1)-C(1)	107.06(12)
C(6)-Pd(1)-Cl(1)	107.71(9)
C(1)-Pd(1)-Cl(1)	144.53(10)
C(6)-Pd(1)-C(2)	135.88(12)
C(1)-Pd(1)-C(2)	35.72(13)
Cl(1)-Pd(1)-C(2)	114.29(8)
C(6)-Pd(1)-Pd(2)	164.89(8)
C(1)-Pd(1)-Pd(2)	86.99(9)
Cl(1)-Pd(1)-Pd(2)	57.76(2)
C(2)-Pd(1)-Pd(2)	59.21(8)
C(9)-Pd(2)-C(3)	101.46(13)
C(9)-Pd(2)-Cl(1)	114.14(9)
C(3)-Pd(2)-Cl(1)	144.40(10)
C(9)-Pd(2)-C(2)	128.37(11)
C(3)-Pd(2)-C(2)	35.33(14)
Cl(1)-Pd(2)-C(2)	112.98(8)
C(9)-Pd(2)-Pd(1)	171.12(9)
C(3)-Pd(2)-Pd(1)	86.85(10)
Cl(1)-Pd(2)-Pd(1)	57.60(2)
C(2)-Pd(2)-Pd(1)	58.00(8)
Pd(1)-Cl(1)-Pd(2)	64.64(2)
C(6)-N(1)-C(7)	111.5(3)
C(6)-N(1)-C(12)	124.6(3)
C(7)-N(1)-C(12)	123.6(3)

C(6)-N(2)-C(8)	111.3(3)
C(6)-N(2)-C(24)	123.8(3)
C(8)-N(2)-C(24)	123.6(3)
C(9)-N(3)-C(10)	111.5(3)
C(9)-N(3)-C(36)	124.3(3)
C(10)-N(3)-C(36)	124.0(3)
C(9)-N(4)-C(11)	112.0(3)
C(9)-N(4)-C(48)	123.4(3)
C(11)-N(4)-C(48)	123.9(3)
C(2)-C(1)-C(5)	105.4(3)
C(2)-C(1)-Pd(1)	85.0(2)
C(5)-C(1)-Pd(1)	103.6(2)
C(2)-C(1)-H(1)	122(3)
C(5)-C(1)-H(1)	124(3)
Pd(1)-C(1)-H(1)	108(2)
C(1)-C(2)-C(3)	109.4(4)
C(1)-C(2)-Pd(1)	59.28(17)
C(3)-C(2)-Pd(1)	109.3(2)
C(1)-C(2)-Pd(2)	108.6(2)
C(3)-C(2)-Pd(2)	57.46(17)
Pd(1)-C(2)-Pd(2)	62.79(8)
C(1)-C(2)-H(2)	124(3)
C(3)-C(2)-H(2)	125(3)
Pd(1)-C(2)-H(2)	108(3)
Pd(2)-C(2)-H(2)	111(3)
C(2)-C(3)-C(4)	105.6(3)
C(2)-C(3)-Pd(2)	87.2(2)
C(4)-C(3)-Pd(2)	102.0(2)
C(2)-C(3)-H(3)	127(3)
C(4)-C(3)-H(3)	120(3)
Pd(2)-C(3)-H(3)	107(3)
C(5)-C(4)-C(3)	109.4(4)
C(5)-C(4)-H(4)	125.3
C(3)-C(4)-H(4)	125.3
C(4)-C(5)-C(1)	110.2(4)
C(4)-C(5)-H(5)	124.9

C(1)-C(5)-H(5)	124.9
N(1)-C(6)-N(2)	103.3(3)
N(1)-C(6)-Pd(1)	130.9(3)
N(2)-C(6)-Pd(1)	125.3(2)
C(8)-C(7)-N(1)	106.7(3)
C(8)-C(7)-H(7)	126.7
N(1)-C(7)-H(7)	126.7
C(7)-C(8)-N(2)	107.2(3)
C(7)-C(8)-H(8)	126.4
N(2)-C(8)-H(8)	126.4
N(3)-C(9)-N(4)	103.2(3)
N(3)-C(9)-Pd(2)	134.2(3)
N(4)-C(9)-Pd(2)	122.3(3)
C(11)-C(10)-N(3)	107.0(3)
C(11)-C(10)-H(10)	126.5
N(3)-C(10)-H(10)	126.5
C(10)-C(11)-N(4)	106.3(3)
C(10)-C(11)-H(11)	126.8
N(4)-C(11)-H(11)	126.8
C(13)-C(12)-C(17)	122.7(3)
C(13)-C(12)-N(1)	119.5(3)
C(17)-C(12)-N(1)	117.8(3)
C(14)-C(13)-C(12)	117.3(3)
C(14)-C(13)-C(18)	119.8(3)
C(12)-C(13)-C(18)	122.8(3)
C(15)-C(14)-C(13)	121.6(4)
C(15)-C(14)-H(14)	119.2
C(13)-C(14)-H(14)	119.2
C(14)-C(15)-C(16)	119.8(4)
C(14)-C(15)-H(15)	120.1
C(16)-C(15)-H(15)	120.1
C(15)-C(16)-C(17)	121.5(3)
C(15)-C(16)-H(16)	119.2
C(17)-C(16)-H(16)	119.2
C(16)-C(17)-C(12)	117.1(3)
C(16)-C(17)-C(21)	120.4(3)

C(12)-C(17)-C(21)	122.5(3)
C(13)-C(18)-C(19)	111.3(3)
C(13)-C(18)-C(20)	110.5(3)
C(19)-C(18)-C(20)	110.6(3)
C(13)-C(18)-H(18)	108.1
C(19)-C(18)-H(18)	108.1
C(20)-C(18)-H(18)	108.1
C(18)-C(19)-H(19A)	109.5
C(18)-C(19)-H(19B)	109.5
H(19A)-C(19)-H(19B)	109.5
C(18)-C(19)-H(19C)	109.5
H(19A)-C(19)-H(19C)	109.5
H(19B)-C(19)-H(19C)	109.5
C(18)-C(20)-H(20A)	109.5
C(18)-C(20)-H(20B)	109.5
H(20A)-C(20)-H(20B)	109.5
C(18)-C(20)-H(20C)	109.5
H(20A)-C(20)-H(20C)	109.5
H(20B)-C(20)-H(20C)	109.5
C(23)-C(21)-C(17)	113.3(3)
C(23)-C(21)-C(22)	109.8(3)
C(17)-C(21)-C(22)	109.7(3)
C(23)-C(21)-H(21)	108.0
C(17)-C(21)-H(21)	108.0
C(22)-C(21)-H(21)	108.0
C(21)-C(22)-H(22A)	109.5
C(21)-C(22)-H(22B)	109.5
H(22A)-C(22)-H(22B)	109.5
C(21)-C(22)-H(22C)	109.5
H(22A)-C(22)-H(22C)	109.5
H(22B)-C(22)-H(22C)	109.5
C(21)-C(23)-H(23A)	109.5
C(21)-C(23)-H(23B)	109.5
H(23A)-C(23)-H(23B)	109.5
C(21)-C(23)-H(23C)	109.5
H(23A)-C(23)-H(23C)	109.5

H(23B)-C(23)-H(23C)	109.5
C(29)-C(24)-C(25)	122.8(4)
C(29)-C(24)-N(2)	117.6(3)
C(25)-C(24)-N(2)	119.6(3)
C(26)-C(25)-C(24)	117.0(4)
C(26)-C(25)-C(30)	121.6(3)
C(24)-C(25)-C(30)	121.4(4)
C(25)-C(26)-C(27)	121.6(4)
C(25)-C(26)-H(26)	119.2
C(27)-C(26)-H(26)	119.2
C(26)-C(27)-C(28)	120.4(4)
C(26)-C(27)-H(27)	119.8
C(28)-C(27)-H(27)	119.8
C(27)-C(28)-C(29)	120.9(4)
C(27)-C(28)-H(28)	119.5
C(29)-C(28)-H(28)	119.5
C(28)-C(29)-C(24)	117.3(3)
C(28)-C(29)-C(33)	120.2(4)
C(24)-C(29)-C(33)	122.4(4)
C(25)-C(30)-C(32)	112.9(3)
C(25)-C(30)-C(31)	111.7(3)
C(32)-C(30)-C(31)	109.4(3)
C(25)-C(30)-H(30)	107.5
C(32)-C(30)-H(30)	107.5
C(31)-C(30)-H(30)	107.5
C(30)-C(31)-H(31A)	109.5
C(30)-C(31)-H(31B)	109.5
H(31A)-C(31)-H(31B)	109.5
C(30)-C(31)-H(31C)	109.5
H(31A)-C(31)-H(31C)	109.5
H(31B)-C(31)-H(31C)	109.5
C(30)-C(32)-H(32A)	109.5
C(30)-C(32)-H(32B)	109.5
H(32A)-C(32)-H(32B)	109.5
C(30)-C(32)-H(32C)	109.5
H(32A)-C(32)-H(32C)	109.5

H(32B)-C(32)-H(32C)	109.5
C(29)-C(33)-C(35)	110.8(3)
C(29)-C(33)-C(34)	112.5(3)
C(35)-C(33)-C(34)	110.9(3)
C(29)-C(33)-H(33)	107.5
C(35)-C(33)-H(33)	107.5
C(34)-C(33)-H(33)	107.5
C(33)-C(34)-H(34A)	109.5
C(33)-C(34)-H(34B)	109.5
H(34A)-C(34)-H(34B)	109.5
C(33)-C(34)-H(34C)	109.5
H(34A)-C(34)-H(34C)	109.5
H(34B)-C(34)-H(34C)	109.5
C(33)-C(35)-H(35A)	109.5
C(33)-C(35)-H(35B)	109.5
H(35A)-C(35)-H(35B)	109.5
C(33)-C(35)-H(35C)	109.5
H(35A)-C(35)-H(35C)	109.5
H(35B)-C(35)-H(35C)	109.5
C(41)-C(36)-C(37)	122.6(3)
C(41)-C(36)-N(3)	119.0(3)
C(37)-C(36)-N(3)	118.4(3)
C(38)-C(37)-C(36)	117.2(3)
C(38)-C(37)-C(42)	120.0(3)
C(36)-C(37)-C(42)	122.8(3)
C(39)-C(38)-C(37)	121.0(4)
C(39)-C(38)-H(38)	119.5
C(37)-C(38)-H(38)	119.5
C(40)-C(39)-C(38)	120.2(4)
C(40)-C(39)-H(39)	119.9
C(38)-C(39)-H(39)	119.9
C(39)-C(40)-C(41)	121.5(3)
C(39)-C(40)-H(40)	119.2
C(41)-C(40)-H(40)	119.2
C(40)-C(41)-C(36)	117.4(4)
C(40)-C(41)-C(45)	120.7(3)

C(36)-C(41)-C(45)	121.9(4)
C(37)-C(42)-C(44)	110.5(3)
C(37)-C(42)-C(43)	111.4(3)
C(44)-C(42)-C(43)	110.9(3)
C(37)-C(42)-H(42)	107.9
C(44)-C(42)-H(42)	107.9
C(43)-C(42)-H(42)	107.9
C(42)-C(43)-H(43A)	109.5
C(42)-C(43)-H(43B)	109.5
H(43A)-C(43)-H(43B)	109.5
C(42)-C(43)-H(43C)	109.5
H(43A)-C(43)-H(43C)	109.5
H(43B)-C(43)-H(43C)	109.5
C(42)-C(44)-H(44A)	109.5
C(42)-C(44)-H(44B)	109.5
H(44A)-C(44)-H(44B)	109.5
C(42)-C(44)-H(44C)	109.5
H(44A)-C(44)-H(44C)	109.5
H(44B)-C(44)-H(44C)	109.5
C(41)-C(45)-C(47)	112.9(3)
C(41)-C(45)-C(46)	111.1(3)
C(47)-C(45)-C(46)	109.2(3)
C(41)-C(45)-H(45)	107.8
C(47)-C(45)-H(45)	107.8
C(46)-C(45)-H(45)	107.8
C(45)-C(46)-H(46A)	109.5
C(45)-C(46)-H(46B)	109.5
H(46A)-C(46)-H(46B)	109.5
C(45)-C(46)-H(46C)	109.5
H(46A)-C(46)-H(46C)	109.5
H(46B)-C(46)-H(46C)	109.5
C(45)-C(47)-H(47A)	109.5
C(45)-C(47)-H(47B)	109.5
H(47A)-C(47)-H(47B)	109.5
C(45)-C(47)-H(47C)	109.5
H(47A)-C(47)-H(47C)	109.5

H(47B)-C(47)-H(47C)	109.5
C(53)-C(48)-C(49)	123.2(3)
C(53)-C(48)-N(4)	119.4(3)
C(49)-C(48)-N(4)	117.4(3)
C(50)-C(49)-C(48)	116.9(4)
C(50)-C(49)-C(54)	120.9(3)
C(48)-C(49)-C(54)	122.1(3)
C(51)-C(50)-C(49)	121.5(3)
C(51)-C(50)-H(50)	119.3
C(49)-C(50)-H(50)	119.3
C(50)-C(51)-C(52)	120.2(4)
C(50)-C(51)-H(51)	119.9
C(52)-C(51)-H(51)	119.9
C(51)-C(52)-C(53)	121.2(4)
C(51)-C(52)-H(52)	119.4
C(53)-C(52)-H(52)	119.4
C(52)-C(53)-C(48)	117.0(3)
C(52)-C(53)-C(57)	121.5(3)
C(48)-C(53)-C(57)	121.5(3)
C(49)-C(54)-C(55)	110.8(3)
C(49)-C(54)-C(56)	113.5(4)
C(55)-C(54)-C(56)	110.0(3)
C(49)-C(54)-H(54)	107.4
C(55)-C(54)-H(54)	107.4
C(56)-C(54)-H(54)	107.4
C(54)-C(55)-H(55A)	109.5
C(54)-C(55)-H(55B)	109.5
H(55A)-C(55)-H(55B)	109.5
C(54)-C(55)-H(55C)	109.5
H(55A)-C(55)-H(55C)	109.5
H(55B)-C(55)-H(55C)	109.5
C(54)-C(56)-H(56A)	109.5
C(54)-C(56)-H(56B)	109.5
H(56A)-C(56)-H(56B)	109.5
C(54)-C(56)-H(56C)	109.5
H(56A)-C(56)-H(56C)	109.5

H(56B)-C(56)-H(56C)	109.5
C(53)-C(57)-C(59)	110.8(3)
C(53)-C(57)-C(58)	112.5(3)
C(59)-C(57)-C(58)	110.5(3)
C(53)-C(57)-H(57)	107.6
C(59)-C(57)-H(57)	107.6
C(58)-C(57)-H(57)	107.6
C(57)-C(58)-H(58A)	109.5
C(57)-C(58)-H(58B)	109.5
H(58A)-C(58)-H(58B)	109.5
C(57)-C(58)-H(58C)	109.5
H(58A)-C(58)-H(58C)	109.5
H(58B)-C(58)-H(58C)	109.5
C(57)-C(59)-H(59A)	109.5
C(57)-C(59)-H(59B)	109.5
H(59A)-C(59)-H(59B)	109.5
C(57)-C(59)-H(59C)	109.5
H(59A)-C(59)-H(59C)	109.5
H(59B)-C(59)-H(59C)	109.5

Table S4: Anisotropic displacement parameters (Å^2× 10^3) for **Cp^{Dim}**. The anisotropic displacement factor exponent takes the form: -2p^2[h^2 a*^{2}U^{11} + ... + 2 h k a* b* U^{12}]

	U^{11}	U^{22}	U^{33}	U^{23}	U^{13}	U^{12}
Pd(1)	21(1)	21(1)	12(1)	-1(1)	5(1)	-2(1)
Pd(2)	22(1)	22(1)	12(1)	-2(1)	5(1)	-4(1)
Cl(1)	28(1)	55(1)	15(1)	-5(1)	4(1)	-20(1)
N(1)	18(1)	24(2)	12(1)	-1(1)	2(1)	-1(1)
N(2)	19(1)	21(2)	16(1)	1(1)	3(1)	-2(1)
N(3)	20(1)	19(2)	13(1)	1(1)	5(1)	0(1)
N(4)	19(1)	21(2)	13(1)	0(1)	2(1)	2(1)
C(1)	19(2)	39(2)	11(2)	-2(2)	-1(1)	-5(2)
C(2)	22(2)	32(2)	20(2)	1(2)	-1(1)	4(2)
C(3)	18(2)	39(2)	16(2)	-3(2)	3(1)	-2(2)
C(4)	21(2)	33(2)	22(2)	-2(2)	-1(1)	-5(2)
C(5)	22(2)	35(2)	20(2)	-8(2)	0(1)	-5(2)
C(6)	18(2)	20(2)	13(2)	1(1)	-3(1)	-1(1)
C(7)	19(2)	32(2)	16(2)	1(2)	6(1)	-3(2)
C(8)	18(2)	29(2)	18(2)	5(2)	6(1)	-3(2)
C(9)	15(1)	19(2)	16(2)	-1(1)	-1(1)	3(1)
C(10)	26(2)	26(2)	12(2)	4(1)	7(1)	4(2)
C(11)	26(2)	28(2)	10(1)	-1(1)	5(1)	3(2)
C(12)	19(2)	26(2)	14(2)	-1(1)	4(1)	-2(1)
C(13)	22(2)	23(2)	16(2)	-1(1)	6(1)	1(2)
C(14)	24(2)	20(2)	20(2)	0(2)	6(1)	2(1)
C(15)	24(2)	23(2)	22(2)	-5(2)	5(1)	-3(2)
C(16)	27(2)	31(2)	14(2)	-3(2)	0(1)	0(2)
C(17)	25(2)	23(2)	16(2)	1(2)	2(1)	1(2)
C(18)	25(2)	23(2)	18(2)	-1(2)	1(1)	3(2)
C(19)	29(2)	43(3)	22(2)	-3(2)	-2(2)	10(2)
C(20)	37(2)	32(2)	17(2)	2(2)	2(2)	6(2)
C(21)	41(2)	24(2)	19(2)	1(2)	-3(1)	-4(2)
C(22)	43(2)	41(3)	23(2)	9(2)	-1(2)	-16(2)
C(23)	49(2)	37(3)	23(2)	5(2)	-2(2)	12(2)
C(24)	25(2)	19(2)	18(2)	0(1)	4(1)	-5(2)

C(25)	28(2)	26(2)	21(2)	2(2)	2(1)	-7(2)
C(26)	47(2)	28(2)	23(2)	-4(2)	-1(2)	-9(2)
C(27)	60(3)	22(2)	37(2)	-5(2)	8(2)	0(2)
C(28)	37(2)	23(2)	35(2)	3(2)	3(2)	4(2)
C(29)	28(2)	23(2)	25(2)	4(2)	5(2)	-1(2)
C(30)	27(2)	29(2)	21(2)	0(2)	-1(1)	-6(2)
C(31)	29(2)	53(3)	24(2)	5(2)	0(2)	-4(2)
C(32)	30(2)	38(3)	20(2)	2(2)	-3(2)	-8(2)
C(33)	26(2)	23(2)	28(2)	4(2)	-2(2)	-1(2)
C(34)	45(2)	49(3)	31(2)	12(2)	-2(2)	-18(2)
C(35)	29(2)	45(3)	43(2)	13(2)	3(2)	-2(2)
C(36)	22(2)	21(2)	12(2)	2(1)	4(1)	-1(1)
C(37)	23(2)	24(2)	15(2)	3(2)	5(1)	3(2)
C(38)	32(2)	23(2)	23(2)	2(2)	3(2)	7(2)
C(39)	35(2)	22(2)	25(2)	-3(2)	1(2)	0(2)
C(40)	24(2)	25(2)	25(2)	2(2)	-2(1)	-4(2)
C(41)	24(2)	28(2)	17(2)	3(2)	6(1)	1(2)
C(42)	21(2)	29(2)	24(2)	2(2)	4(2)	2(2)
C(43)	27(2)	47(3)	30(2)	11(2)	1(2)	4(2)
C(44)	29(2)	55(3)	35(2)	5(2)	12(2)	4(2)
C(45)	22(2)	22(2)	32(2)	3(2)	1(2)	-1(1)
C(46)	33(2)	36(3)	46(2)	3(2)	17(2)	7(2)
C(47)	27(2)	33(3)	43(2)	2(2)	-7(2)	4(2)
C(48)	24(2)	21(2)	13(2)	-7(1)	1(1)	-3(2)
C(49)	23(2)	23(2)	22(2)	-5(2)	2(1)	0(2)
C(50)	20(2)	36(2)	24(2)	-4(2)	-4(1)	-3(2)
C(51)	32(2)	32(2)	33(2)	-2(2)	1(2)	-14(2)
C(52)	36(2)	22(2)	25(2)	3(2)	-4(2)	-4(2)
C(53)	29(2)	20(2)	16(2)	-1(1)	0(1)	-1(2)
C(54)	24(2)	32(2)	30(2)	0(2)	-6(1)	2(2)
C(55)	75(3)	58(4)	27(2)	-3(2)	-5(2)	42(3)
C(56)	70(3)	46(3)	25(2)	6(2)	5(2)	20(3)
C(57)	28(2)	22(2)	19(2)	-1(2)	-5(1)	-1(2)
C(58)	34(2)	41(3)	19(2)	3(2)	-5(2)	4(2)
C(59)	24(2)	45(3)	24(2)	-2(2)	-2(2)	-2(2)

Table S5: Hydrogen coordinates (× 10^4) and isotropic displacement parameters ($Å^2$× 10^{3}) for **Cp^Dim^**.

	x	y	z	U(eq)
H(1)	4340(30)	5160(40)	4360(20)	28
H(2)	5070(40)	4180(40)	3420(20)	30
H(3)	5750(40)	5620(40)	2670(20)	29
H(4)	4955	7414	2950	31
H(5)	4052	7163	3986	32
H(7)	-39	5235	5109	26
H(8)	-533	3471	4525	26
H(10)	5489	6074	30	25
H(11)	4489	4305	-204	26
H(14)	2051	8894	4347	26
H(15)	3409	8852	5388	27
H(16)	3787	7236	6011	30
H(18)	307	6638	3567	26
H(19A)	-633	8046	4084	48
H(19B)	-809	8223	3250	48
H(19C)	135	8934	3767	48
H(20A)	1746	8285	3099	43
H(20B)	739	7636	2587	43
H(20C)	1880	7008	2981	43
H(21)	2506	4630	5475	35
H(22A)	2787	5853	6757	55
H(22B)	2403	4605	6668	55
H(22C)	1532	5536	6308	55
H(23A)	4539	4929	5540	56
H(23B)	4293	4276	6215	56
H(23C)	4595	5544	6275	56
H(26)	-190	1437	2196	40
H(27)	1245	218	2694	48
H(28)	2457	605	3763	39
H(30)	-443	4156	2737	31

H(31A)	-2188	2543	2834	54
H(31B)	-2501	3785	2643	54
H(31C)	-1763	3476	3400	54
H(32A)	-303	3444	1621	46
H(32B)	-1597	3902	1618	46
H(32C)	-1357	2622	1665	46
H(33)	2304	3033	4787	31
H(34A)	1699	1424	5257	64
H(34B)	3038	1660	5603	64
H(34C)	2731	761	4999	64
H(35A)	4124	1795	4350	59
H(35B)	4312	2722	4940	59
H(35C)	3843	3034	4134	59
H(38)	4182	9522	1501	31
H(39)	6123	9788	2049	34
H(40)	7473	8389	2112	31
H(42)	3001	7049	654	29
H(43A)	2737	9352	482	53
H(43B)	1912	8460	36	53
H(43C)	3251	8580	-56	53
H(44A)	2494	7280	1767	58
H(44B)	1459	7741	1177	58
H(44C)	2353	8561	1641	58
H(45)	6833	5644	1584	31
H(46A)	7422	6428	587	55
H(46B)	8513	5833	1064	55
H(46C)	8402	7128	1085	55
H(47A)	8660	6936	2373	54
H(47B)	8571	5639	2379	54
H(47C)	7681	6356	2731	54
H(50)	482	2939	142	33
H(51)	1023	1435	842	40
H(52)	2878	1337	1545	35
H(54)	2337	5256	-1	36
H(55A)	-20	5007	209	82
H(55B)	627	6125	98	82

H(55C)	1003	5452	812	82
H(56A)	1747	4176	-1016	71
H(56B)	961	5251	-1051	71
H(56C)	445	4111	-850	71
H(57)	4983	3481	1808	29
H(58A)	4092	2478	2633	49
H(58B)	5465	2230	2710	49
H(58C)	4522	1377	2321	49
H(59A)	5342	1414	1208	48
H(59B)	6365	2185	1605	48
H(59C)	5596	2556	870	48

Table S6: Torsion angles [°] for **Cp^{Dim}**.

C(5)-C(1)-C(2)-C(3)	-1.3(4)
Pd(1)-C(1)-C(2)-C(3)	101.5(3)
C(5)-C(1)-C(2)-Pd(1)	-102.8(3)
C(5)-C(1)-C(2)-Pd(2)	-62.4(3)
Pd(1)-C(1)-C(2)-Pd(2)	40.4(2)
C(1)-C(2)-C(3)-C(4)	1.4(4)
Pd(1)-C(2)-C(3)-C(4)	64.6(3)
Pd(2)-C(2)-C(3)-C(4)	101.6(3)
C(1)-C(2)-C(3)-Pd(2)	-100.2(3)
Pd(1)-C(2)-C(3)-Pd(2)	-37.0(2)
C(2)-C(3)-C(4)-C(5)	-1.0(4)
Pd(2)-C(3)-C(4)-C(5)	89.4(3)
C(3)-C(4)-C(5)-C(1)	0.2(4)
C(2)-C(1)-C(5)-C(4)	0.6(4)
Pd(1)-C(1)-C(5)-C(4)	-87.9(3)
C(7)-N(1)-C(6)-N(2)	1.2(3)
C(12)-N(1)-C(6)-N(2)	174.4(3)
C(7)-N(1)-C(6)-Pd(1)	173.5(2)
C(12)-N(1)-C(6)-Pd(1)	-13.3(5)
C(8)-N(2)-C(6)-N(1)	-1.3(3)
C(24)-N(2)-C(6)-N(1)	-169.2(3)
C(8)-N(2)-C(6)-Pd(1)	-174.2(2)
C(24)-N(2)-C(6)-Pd(1)	17.9(4)
C(6)-N(1)-C(7)-C(8)	-0.6(4)
C(12)-N(1)-C(7)-C(8)	-173.9(3)
N(1)-C(7)-C(8)-N(2)	-0.3(4)
C(6)-N(2)-C(8)-C(7)	1.0(4)
C(24)-N(2)-C(8)-C(7)	169.0(3)
C(10)-N(3)-C(9)-N(4)	0.1(4)
C(36)-N(3)-C(9)-N(4)	175.1(3)
C(10)-N(3)-C(9)-Pd(2)	173.8(2)
C(36)-N(3)-C(9)-Pd(2)	-11.3(5)
C(11)-N(4)-C(9)-N(3)	-0.1(3)
C(48)-N(4)-C(9)-N(3)	-171.0(3)

C(11)-N(4)-C(9)-Pd(2)	-174.7(2)
C(48)-N(4)-C(9)-Pd(2)	14.4(4)
C(9)-N(3)-C(10)-C(11)	-0.1(4)
C(36)-N(3)-C(10)-C(11)	-175.1(3)
N(3)-C(10)-C(11)-N(4)	0.1(3)
C(9)-N(4)-C(11)-C(10)	0.0(4)
C(48)-N(4)-C(11)-C(10)	170.9(3)
C(6)-N(1)-C(12)-C(13)	83.8(4)
C(7)-N(1)-C(12)-C(13)	-103.8(4)
C(6)-N(1)-C(12)-C(17)	-97.4(4)
C(7)-N(1)-C(12)-C(17)	75.0(4)
C(17)-C(12)-C(13)-C(14)	0.8(5)
N(1)-C(12)-C(13)-C(14)	179.4(3)
C(17)-C(12)-C(13)-C(18)	-179.4(3)
N(1)-C(12)-C(13)-C(18)	-0.7(5)
C(12)-C(13)-C(14)-C(15)	-0.5(5)
C(18)-C(13)-C(14)-C(15)	179.7(3)
C(13)-C(14)-C(15)-C(16)	0.0(5)
C(14)-C(15)-C(16)-C(17)	0.2(5)
C(15)-C(16)-C(17)-C(12)	0.1(5)
C(15)-C(16)-C(17)-C(21)	-178.7(3)
C(13)-C(12)-C(17)-C(16)	-0.6(5)
N(1)-C(12)-C(17)-C(16)	-179.3(3)
C(13)-C(12)-C(17)-C(21)	178.2(3)
N(1)-C(12)-C(17)-C(21)	-0.5(5)
C(14)-C(13)-C(18)-C(19)	-55.9(4)
C(12)-C(13)-C(18)-C(19)	124.3(4)
C(14)-C(13)-C(18)-C(20)	67.4(4)
C(12)-C(13)-C(18)-C(20)	-112.4(4)
C(16)-C(17)-C(21)-C(23)	-51.6(5)
C(12)-C(17)-C(21)-C(23)	129.7(4)
C(16)-C(17)-C(21)-C(22)	71.5(4)
C(12)-C(17)-C(21)-C(22)	-107.2(4)
C(6)-N(2)-C(24)-C(29)	77.2(4)
C(8)-N(2)-C(24)-C(29)	-89.2(4)
C(6)-N(2)-C(24)-C(25)	-104.3(4)

C(8)-N(2)-C(24)-C(25)	89.3(4)
C(29)-C(24)-C(25)-C(26)	-0.7(5)
N(2)-C(24)-C(25)-C(26)	-179.1(3)
C(29)-C(24)-C(25)-C(30)	179.4(3)
N(2)-C(24)-C(25)-C(30)	1.0(5)
C(24)-C(25)-C(26)-C(27)	-0.9(6)
C(30)-C(25)-C(26)-C(27)	179.0(4)
C(25)-C(26)-C(27)-C(28)	1.2(7)
C(26)-C(27)-C(28)-C(29)	0.1(7)
C(27)-C(28)-C(29)-C(24)	-1.5(6)
C(27)-C(28)-C(29)-C(33)	-179.2(4)
C(25)-C(24)-C(29)-C(28)	1.8(5)
N(2)-C(24)-C(29)-C(28)	-179.7(3)
C(25)-C(24)-C(29)-C(33)	179.5(3)
N(2)-C(24)-C(29)-C(33)	-2.0(5)
C(26)-C(25)-C(30)-C(32)	-29.2(5)
C(24)-C(25)-C(30)-C(32)	150.8(3)
C(26)-C(25)-C(30)-C(31)	94.6(4)
C(24)-C(25)-C(30)-C(31)	-85.5(4)
C(28)-C(29)-C(33)-C(35)	66.7(5)
C(24)-C(29)-C(33)-C(35)	-110.8(4)
C(28)-C(29)-C(33)-C(34)	-58.0(5)
C(24)-C(29)-C(33)-C(34)	124.4(4)
C(9)-N(3)-C(36)-C(41)	106.8(4)
C(10)-N(3)-C(36)-C(41)	-78.8(4)
C(9)-N(3)-C(36)-C(37)	-74.0(4)
C(10)-N(3)-C(36)-C(37)	100.3(4)
C(41)-C(36)-C(37)-C(38)	-1.4(5)
N(3)-C(36)-C(37)-C(38)	179.6(3)
C(41)-C(36)-C(37)-C(42)	179.9(3)
N(3)-C(36)-C(37)-C(42)	0.8(5)
C(36)-C(37)-C(38)-C(39)	0.9(5)
C(42)-C(37)-C(38)-C(39)	179.8(3)
C(37)-C(38)-C(39)-C(40)	-0.3(6)
C(38)-C(39)-C(40)-C(41)	0.0(6)
C(39)-C(40)-C(41)-C(36)	-0.3(5)

C(39)-C(40)-C(41)-C(45)	179.2(3)
C(37)-C(36)-C(41)-C(40)	1.1(5)
N(3)-C(36)-C(41)-C(40)	-179.9(3)
C(37)-C(36)-C(41)-C(45)	-178.4(3)
N(3)-C(36)-C(41)-C(45)	0.6(5)
C(38)-C(37)-C(42)-C(44)	-66.9(5)
C(36)-C(37)-C(42)-C(44)	111.9(4)
C(38)-C(37)-C(42)-C(43)	56.9(4)
C(36)-C(37)-C(42)-C(43)	-124.3(4)
C(40)-C(41)-C(45)-C(47)	39.6(5)
C(36)-C(41)-C(45)-C(47)	-140.9(4)
C(40)-C(41)-C(45)-C(46)	-83.5(4)
C(36)-C(41)-C(45)-C(46)	96.0(4)
C(9)-N(4)-C(48)-C(53)	-80.8(4)
C(11)-N(4)-C(48)-C(53)	109.4(4)
C(9)-N(4)-C(48)-C(49)	97.2(4)
C(11)-N(4)-C(48)-C(49)	-72.7(4)
C(53)-C(48)-C(49)-C(50)	-2.2(5)
N(4)-C(48)-C(49)-C(50)	179.9(3)
C(53)-C(48)-C(49)-C(54)	173.7(3)
N(4)-C(48)-C(49)-C(54)	-4.2(5)
C(48)-C(49)-C(50)-C(51)	1.0(5)
C(54)-C(49)-C(50)-C(51)	-175.0(4)
C(49)-C(50)-C(51)-C(52)	0.8(6)
C(50)-C(51)-C(52)-C(53)	-1.4(6)
C(51)-C(52)-C(53)-C(48)	0.2(6)
C(51)-C(52)-C(53)-C(57)	179.1(4)
C(49)-C(48)-C(53)-C(52)	1.7(5)
N(4)-C(48)-C(53)-C(52)	179.5(3)
C(49)-C(48)-C(53)-C(57)	-177.3(3)
N(4)-C(48)-C(53)-C(57)	0.6(5)
C(50)-C(49)-C(54)-C(55)	66.6(5)
C(48)-C(49)-C(54)-C(55)	-109.1(4)
C(50)-C(49)-C(54)-C(56)	-57.7(5)
C(48)-C(49)-C(54)-C(56)	126.6(4)
C(52)-C(53)-C(57)-C(59)	91.1(4)

C(48)-C(53)-C(57)-C(59)	-90.0(4)
C(52)-C(53)-C(57)-C(58)	-33.1(5)
C(48)-C(53)-C(57)-C(58)	145.8(4)
